# Laryngeal tuberculosis in renal transplant recipients: A case report and review of the literature

**DOI:** 10.17305/bjbms.2020.4448

**Published:** 2020-08

**Authors:** Fabrizio Cialente, Michele Grasso, Massimo Ralli, Marco de Vincentiis, Antonio Minni, Griselda Agolli, Michele Dello Spedale Venti, Mara Riminucci, Alessandro Corsi, Antonio Greco

**Affiliations:** 1 Department of Sense Organs, Sapienza University of Rome, Rome, Italy; 2 Department of Oral and Maxillofacial Sciences, Sapienza University of Rome, Rome, Italy; 3 Department of Molecular Medicine, Sapienza University of Rome, Rome, Italy

**Keywords:** Laryngeal tuberculosis, kidney transplant, laryngeal leukoplakia

## Abstract

Renal allograft recipients are at greater risk of developing tuberculosis than the general population. A woman with a kidney transplant was admitted to our emergency department with high temperature, dysphonia, odynophagia, and asthenia. The final diagnosis was laryngeal tuberculosis. Multidisciplinary collaboration enabled accurate diagnosis and successful treatment. Laryngeal tuberculosis should be considered in renal allograft recipients with hoarseness. A more rapid diagnosis of tuberculosis in renal transplant recipients is desirable when the site involved, such as the larynx, exhibits specific manifestations and the patient exhibits specific symptoms. In these cases, prognosis is excellent, and with adequate treatment a complete recovery is often achieved.

## INTRODUCTION

Tuberculosis (TB) is an infectious disease caused by *Mycobacterium tuberculosis* [1]. Geographically, most TB cases in 2018 were in Southeast Asia (44%), Africa (24%), and Western Pacific nations (18%); smaller numbers of cases have occurred in Eastern Mediterranean nations (8%), the Americas (3%), and Europe (3%) [1]. In 2018, the number of new cases of rifampicin-resistant TB was approximately half a million, of which 78% were multidrug-resistant TB [1]. Although the prevalence of TB is low in Italy, its epidemiology is changing. Since 1955, more than 160,000 people in Italy have died from this potentially preventable and curable disease [2]. In 2018, the estimated incidence of TB in Italy was 4300 new cases (7 cases per 100,000 population), and the estimated number of deaths from TB in Italy was 370. The number of TB case notifications in 2018 in Italy was 3912 (71% with pulmonary involvement) [1].

Although the lung is the most common site of involvement, a number of extrapulmonary organs, including the larynx, can be involved as well. Laryngeal TB is an uncommon condition and has been rarely reported in the literature [1,3]. Nevertheless, it represents a public health concern [4] and may appear similar to malignancy on imaging and laryngoscopy [5,6].

Renal allograft recipients are at greater risk of developing TB, commonly atypical and extrapulmonary, than is the general population. The prevalence of TB in renal transplant recipients ranges from 1% to 4% in developed Western countries [7-10].

We present the case of a renal transplant recipient who developed laryngeal TB. This case represents a rare condition.

## CASE REPORT

A 38-year-old woman with chronic renal failure caused by chronic diabetes mellitus from the age of 9 was admitted to the Emergency Department of the Sapienza University Hospital, Rome, Italy, in December 2018 with high temperature, dysphonia, odynophagia, general discomfort, and severe asthenia. She reported having undergone a thoracotomy with right-sided pneumectomy at the age of 16 for previous pulmonary aspergillosis, and renal transplantation in 2015 at the age of 35 and subsequent treatment with steroids, mycophenolate mofetil, and tacrolimus. Laryngeal and pulmonary mycosis were initially suspected, and she was transferred to the Department of Infectious Diseases and treated with antimycotic drugs.

**FIGURE 1:**
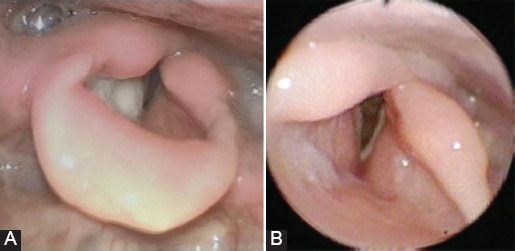
Laryngoscopic images. (A) Before antitubercular therapy. Swelling of the right false vocal fold with a whitish lesion, normal motility, and respiratory space. (B) After antitubercular therapy. Normal motility and structure of larynx, with normal respiratory space.

A neck and pulmonary computed tomographic (CT) scan showed asymmetry of the glottic space with marked contrast enhancement of the epiglottis up to the false right vocal fold; submucosal imbibition up to the thyroid cartilage, which appeared to be inhomogeneous with pseudo-focality; and right-sided Level II lymphadenopathy with a maximum lymph node diameter of 12 mm. We also found right-sided lung atelectasis with ipsilateral mediastinal displacement (as a result of the earlier thoracotomy with right pneumectomy) and hyperplasia of the left lung with interstitial thickening. Otolaryngologic examination with high-definition fiberoptic laryngoscopy (Figure 1) showed swelling of the right false vocal fold with a whitish lesion; motility and respiratory space were normal. Endoscopy of the larynx with narrow-band imaging confirmed that the lesion was not malignant.

**FIGURE 2:**
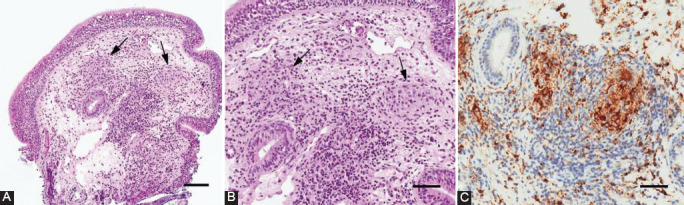
Hematoxylin and eosin staining of the sampled mucosa under low- (A) and high-power (B) magnification illustrates the lymphocyte-rich inflammatory infiltrates along with multiple small, nonnecrotizing granulomas (arrows in A and B). The immunohistochemical stain for CD68R (C) highlights the small granulomatous aggregates of histiocytes. Bars represent 100 µm in A and 50 µm in B and C.

The patient underwent microlaryngoscopy under general anesthesia, and a biopsy sample of the whitish lesion was obtained. Histologic examination (Figure 2) revealed that the mucosa was lined by respiratory epithelium free from atypia. Lymphocyte-rich inflammatory infiltrates, along with multiple small, nonnecrotizing granulomatous aggregates of epithelioid histiocytes (CD68R+), were detected in the lamina propria. Ziehl-Neelsen and periodic acid–Schiff staining did not yield conclusive findings. Subsequent microbiologic bronchoalveolar lavage and sputum tests confirmed the diagnosis of pulmonary TB with extrapulmonary laryngeal extension. The patient was then isolated for 30 days and treated daily with 200 mg of isoniazid, 400 mg of ethambutol, 500 mg of pyrazinamide, 400 mg of moxifloxacin, and 300 mg of Vitamin B_6_ (Benadon). As of this writing, the patient is not isolated and undergoes regular follow-up visits with clinical examinations and CT scans every 2 months.

## DISCUSSION

Renal transplant recipients are at high risk for different infections because they must receive immunosuppressive therapy [11]. Using the keywords “laryngeal TB” and “kidney transplant,” we searched PubMed for articles written in English from 1990 and on about the occurrence of laryngeal TB in renal transplant recipients. The inclusion criteria were kidney transplantation and laryngeal TB, and we included only articles in which laryngeal involvement occurred before or at the same time as pulmonary involvement. We reviewed five reports about a total of eight patients [2,10,12,13]. In these cases, laryngeal symptoms were prominent (dysphonia and hoarseness) and directed the diagnosis. We excluded one article in which the authors diagnosed laryngeal TB after the occurrence of pulmonary TB (in a patient with cavitary pulmonary TB who was found to have laryngeal TB 1 year after discontinuing antituberculous drugs) [7]. In our review, we evaluated the following parameters: clinical manifestations, results of diagnostic tests, and treatment. In these patients (Table 1), the main symptoms were hoarseness, dysphonia, odynophagia, cough and fever, and biopsy samples of tissue revealed granulomatous inflammation. The treatment of TB may be complex, and drug resistance often develops [14,15]. Standard therapeutic agents include isoniazid, rifampicin and pyrazinamide, although modifications of therapy because of cost have been reported in the literature [2]; neither of the patients were given rifampicin because it would have necessitated an increase in the cyclosporine dose and, consequently, the cost of therapy.

**TABLE 1:**
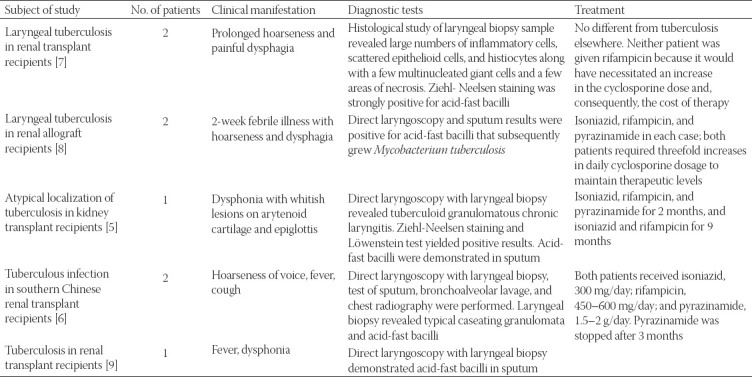
Cases of laryngeal tuberculosis reported in the literature

Since laryngeal TB in renal transplant recipients is an atypical presentation of TB, the diagnosis may be delayed, which can lead to increased morbidity and exposure of the patient’s contacts, particularly through nosocomial transmission. The mechanism of laryngeal involvement is postulated to be through bronchogenic spread, with infected sputum from the lungs directly contaminating the larynx. However, this theory has been challenged by other authors [10], who suggested that hematogenous spread is responsible for laryngeal seeding.

Immunosuppressive drugs promote reactivation of latent TB and acquisition of new disease. In many studies [2,11,12], the main symptoms that forced patients to seek medical attention were severe fever and severe odynophagia, although patients had already had other symptoms, such as dysphonia and asthenia, for weeks. The differential diagnosis must include laryngeal TB. Larynx assessment with advanced endoscopy and narrow-band imaging enables diagnosis [16]. We used endoscopes capable of narrow-band imaging to rule out suspected malignant lesions of the larynx because our institution is a referral center for the treatment of laryngeal cancer in Italy. Whitish lesions of larynx can occur in many types of diseases, ranging from benign hyperkeratosis to squamous cell carcinoma. Other clinical conditions included in the differential diagnosis are sarcoidosis, Wegener’s granulomatosis, syphilis, histoplasmosis, blastomycosis, coccidioidomycosis, cryptococcosis, amyloidosis, traumatic polypoid granulation, and laryngeal papilloma [16-18]. Thus, biopsy may be performed for differential diagnostic purpose.

In two cases in the literature, the standard treatment was isoniazid, rifampicin and pyrazinamide, although both patients required a threefold increase in the daily cyclosporine dosage to maintain therapeutic levels. Because of the drug interaction between rifampicin and cyclosporine, the dosage of cyclosporine must be increased substantially when it is coadministered with rifampicin [19,20]. The results of both randomized and nonrandomized studies support the value of isoniazid as TB prophylaxis in renal transplant recipients at risk for active infection [21]. Clinicians should consider prophylaxis in renal transplant recipients in endemic areas or in recipients in nonendemic countries who are at risk. We treated our patient with isoniazid because she was at risk for active infection, according to the literature [19,22-24]. However, the evidence of the benefit of isoniazid prophylaxis in renal transplantation is not robust, and a large multicenter trial is needed to investigate isoniazid prophylaxis in kidney transplantation in an endemic area [25].

Laryngeal TB is rare in renal transplant recipients who developed TB. The few cases reported in the literature indicate that laryngeal symptoms (dysphonia and dysphagia) in such patients must be assessed adequately, even in industrialized countries in which TB is not widespread. In these cases, prognosis is excellent and recovery is often achieved. When whitish lesions are found in the larynx, diagnostic tests for TB must be performed.

## CONCLUSION

Laryngeal TB should be considered in renal allograft recipients with hoarseness. A more rapid diagnosis of TB in renal transplant recipients is desirable when the site involved, such as the larynx, exhibits specific manifestations and the patient exhibits specific symptoms. In these cases, prognosis is excellent, and with adequate treatment, a complete recovery is often achieved.
